# Comorbid Auditory and Visual Dysfunction: From Pathogenic Genes to Gene Therapy

**DOI:** 10.3390/genes17091149

**Published:** 2026-09-19

**Authors:** Jindie Hu, Chenyang Kong, Yu Sun

**Affiliations:** 1 Department of Otorhinolaryngology, Union Hospital, Tongji Medical College, Huazhong University of Science and Technology, Wuhan 430022, China; 2 Institute of Otorhinolaryngology, Union Hospital, Tongji Medical College, Huazhong University of Science and Technology, Wuhan 430022, China; 3 Hubei Province Clinical Research Center for Deafness and Vertigo, Wuhan 430022, China

**Keywords:** deafness, blindness, pathogenic gene, gene therapy

## Abstract

Hearing and vision are the most important sensory functions. Genetic studies have revealed that specific genetic mutations can concurrently induce auditory and visual dysfunction. Comorbid auditory and visual impairment limits mutual sensory compensation, thereby severely delaying speech, cognitive, and intellectual development in affected pediatric patients and imposing a profound burden on their families. In this review, we summarize 23 genes currently recognized to be associated with both auditory and visual impairment and classify them according to their underlying pathogenic mechanisms. Furthermore, recent advances in gene therapy have created new opportunities for the molecular treatment of inherited auditory and visual disorders, while local gene therapy trials targeting the eye or inner ear have shown encouraging clinical signals. However, most current approaches remain gene- or organ-specific, and several programs are still at the preclinical or early clinical stage. Therefore, we also summarize the latest progress of gene-based therapies and ongoing clinical trial programs targeting hereditary deafness and blindness, aiming to provide references and basis for the subsequent treatment of comorbid auditory and visual dysfunctions.

## 1. Introduction

Hearing and vision are the two principal sensory gateways connecting individuals to the external world and are indispensable for language acquisition, cognitive development, communication, mobility, and independent living. According to the World Health Organization, more than 1.5 billion people worldwide currently live with some degree of hearing loss [1]. The global burden of visual impairment is even greater, with at least 2.2 billion people affected by near- or distance-vision impairment creating a significant public health burden [2].

However, a subset of genetic disorders affects both sensory systems and gives rise to combined auditory and visual impairment, hereafter referred to as hereditary auditory–visual comorbidity [3]. It encompasses classical deaf–blind syndromes, such as Usher syndrome, as well as disorders associated with retinal degeneration, congenital ocular malformation, optic neuropathy, corneal disease, or visual-pathway dysfunction accompanied by hearing loss [4,5]. Dual sensory impairment cannot be regarded simply as the additive effect of isolated deafness and blindness. Because vision often compensates for impaired hearing and hearing compensates for reduced vision, simultaneous dysfunction of both systems markedly restricts access to language, environmental information, interpersonal communication, education, mobility, and social participation [6]. In children with congenital or early-onset disease, inadequate sensory and language access may interfere with speech and cognitive development, whereas affected individuals across the lifespan face increased risks of social isolation, psychological distress, and reduced quality of life [7].

The diagnosis of hereditary auditory–visual disorders is complicated by marked clinical and genetic heterogeneity [8,9,10,11,12]. Hearing loss may be congenital, delayed-onset, stable, or progressive and may arise from abnormalities of cochlear hair cells, the stria vascularis, spiral ganglion neurons, auditory nerves, or middle-ear structures. Similarly, visual manifestations range from retinitis pigmentosa, cone or cone-rod dysfunction, and congenital blindness to optic atrophy, retinal vascular dysplasia, ocular coloboma, corneal dystrophy, and cortical visual impairment [8,13,14,15,16,17]. Furthermore, variants in the same gene may produce isolated hearing loss, isolated ocular disease, or combined auditory–visual involvement depending on the variant type, residual protein function, inheritance pattern, and genetic background [18,19].

**Figure 1:**
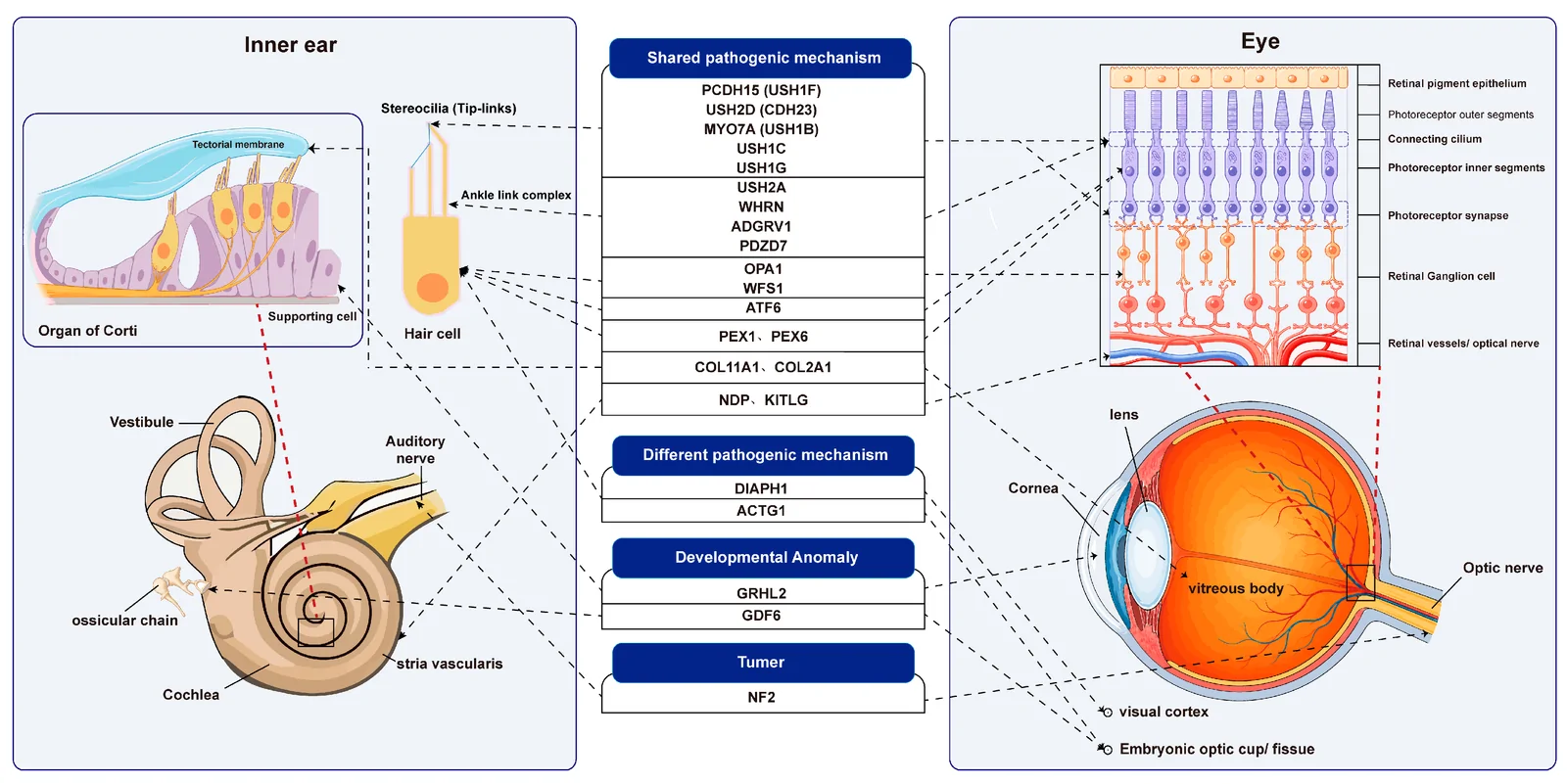
Pathogenic mechanisms of comorbid auditory and visual dysfunction. The central boxes group the genes according to their predominant pathogenic mechanisms. Black dashed lines indicate the principal anatomical or cellular sites associated with each gene or gene group, whereas red dashed lines link the organ-level schematics to the corresponding enlarged structural insets.

Despite this phenotypic diversity, the auditory and visual systems share several molecular and cellular requirements. Cochlear hair cells and retinal photoreceptors are highly specialized sensory cells that depend on precisely organized cytoskeletal structures, ciliary or stereociliary protein complexes, intracellular cargo trafficking, membrane adhesion, synaptic integrity, and tightly regulated metabolic homeostasis, as illustrated in Figure 1 [20,21]. Accordingly, pathogenic mechanisms underlying auditory–visual comorbidity include disruption of Usher adhesion-scaffold complexes, impaired actin-dependent transport, defective ciliary trafficking, mitochondrial or endoplasmic-reticulum dysfunction, peroxisomal biogenesis defects, extracellular-matrix abnormalities, and altered developmental or vascular signaling [22,23,24,25,26,27]. However, not all associated genes act through an identical mechanism in both organs; some affect a shared molecular pathway but different tissue-specific targets, whereas others produce auditory and visual phenotypes through distinct allelic or developmental mechanisms. Importantly, hereditary auditory–visual comorbidity does not invariably result from primary degeneration of sensory cells. In some disorders, combined sensory impairment arises secondarily from structural or tumor-mediated abnormalities that damage or compress the auditory and visual pathways.

A mechanism-based understanding of these disorders is therefore essential for accurate molecular diagnosis, genotype–phenotype interpretation, prognostic counselling, and therapeutic development. Recent advances in gene therapies have provided proof of concept that inherited sensory dysfunction may be treated at its molecular origin. Nevertheless, current therapies generally target either the inner ear or the eye, and simultaneous restoration of hearing and vision remains challenging. In this review, we summarize the major genes and syndromes associated with hereditary auditory–visual comorbidity, classify them according to their shared or organ-specific pathogenic mechanisms, and discuss emerging gene therapeutic strategies.

## 2. Review Methods

### 2.1. Gene Selection

Candidate genes were initially identified using established gene–disease resources, including OMIM and the Hereditary Hearing Loss Homepage, to identify genes with well-supported associations with hereditary hearing loss. These candidates were subsequently evaluated for evidence of ocular or visual involvement, including retinal degeneration, optic neuropathy, ocular developmental defects, corneal disease, and other clinically relevant manifestations affecting the visual system. The selected genes included those causing established syndromic auditory–visual disorders, as well as genes in which different pathogenic variants or allelic contexts have been associated with auditory, visual, or combined auditory–visual phenotypes. Genes for which the gene–disease relationship or the association with combined auditory and visual dysfunction remained uncertain were excluded. On this basis, 23 genes were identified and included for detailed discussion. These genes were subsequently classified according to their predominant pathogenic mechanisms.

### 2.2. Literature Search and Selection

For the 23 selected genes, literature searches were performed using PubMed, Web of Science, and Scopus, covering publications from January 2000 to July 2026. The retrieved literature was summarized and cross-verified, with both comprehensive findings and illustrative studies considered. The search strategy combined individual gene symbols with terms related to auditory and visual phenotypes, molecular mechanisms, and therapeutic development. Representative search combinations included “*USH2A* AND hearing loss,” “*USH2A* AND retinal degeneration,” “*OPA1* AND hearing loss,” and “*OPA1* AND visual impairment.” Peer-reviewed studies were prioritized, while authoritative clinical-trial registries were additionally consulted to verify the status of ongoing therapeutic programs. Non-peer-reviewed secondary sources lacking sufficient supporting evidence were not used as primary evidence.

## 3. Deafness–Blindness Syndromes

### 3.1. Usher Syndrome

Usher syndrome (USH) is an autosomal recessive disorder characterized by the combination of sensorineural hearing loss and progressive retinal degeneration, most commonly retinitis pigmentosa. It represents the leading inherited cause of combined hearing and visual impairment [17,19,23,28]. The two defining sensory abnormalities have different temporal courses. Hearing loss is usually congenital or begins early in life, whereas retinal degeneration typically becomes evident later, initially presenting with nyctalopia and impaired dark adaptation, followed by progressive constriction of the peripheral visual field and, in advanced disease, loss of central vision [5]. Vestibular dysfunction is an additional major phenotypic discriminator and is especially prominent in Usher syndrome type 1. The delayed onset of retinal symptoms means that affected children may initially be diagnosed as having isolated congenital deafness, highlighting the importance of early molecular testing and longitudinal ophthalmologic surveillance [4,18]. Usher syndrome is traditionally divided into three clinical subtypes according to the severity and onset of hearing loss, vestibular function, and the age at onset and progression of retinal degeneration. This clinical classification remains useful, although substantial phenotypic overlap and atypical presentations are increasingly recognized [23].

#### 3.1.1. Usher Syndrome Type 1

Usher syndrome type 1 (USH1) is the most severe classical subtype. It is characterized by congenital bilateral severe-to-profound sensorineural hearing loss, marked vestibular hypofunction or areflexia, and progressive retinitis pigmentosa, usually becoming symptomatic during childhood or adolescence [19,29]. Because of vestibular dysfunction, affected children often exhibit delayed independent walking and impaired balance. Without early auditory rehabilitation, including timely cochlear implantation when appropriate, spoken-language development may be profoundly compromised [30].

Five genes are currently regarded as the principal, well-established causes of classical USH1: *MYO7A*, *USH1C*, *CDH23*, *PCDH15*, and *USH1G* [31]. These genes encode proteins that form an interconnected molecular network in sensory hair cells and retinal photoreceptors. In cochlear hair cells, the USH1 proteins participate in the development, organization, cohesion, and mechanotransduction function of stereociliary bundles [32]. In the retina, the same proteins contribute to photoreceptor ciliary trafficking, synaptic organization, and the functional interaction between photoreceptors and the retinal pigment epithelium. However, their precise retinal localization and functions differ from those in the inner ear. Thus, USH1 proteins act through a shared molecular network in both sensory systems, but the immediate cellular consequences are organ specific. *CIB2* was previously proposed as the cause of an additional USH1 subtype [33]. Subsequent genetic and phenotypic studies, however, have more consistently associated biallelic *CIB2* variants with autosomal recessive nonsyndromic hearing loss, and its status as a definitive Usher syndrome gene remains uncertain.

#### 3.1.2. Usher Syndrome Type 2

Usher syndrome type 2 (USH2) is generally characterized by congenital bilateral moderate-to-severe sensorineural hearing loss, relatively preserved vestibular function, and retinitis pigmentosa that commonly becomes symptomatic in late adolescence or early adulthood [34]. Hearing loss is often greater at higher frequencies and may initially be managed with hearing aids, although progression and severity vary among individuals. Vestibular function is usually normal, but mild or subclinical vestibular abnormalities have been reported in a subset of patients [35].

The three established USH2 genes are *USH2A*, *ADGRV1*, and *WHRN*. These proteins assemble with other components into the USH2 protein complex, which is localized to the ankle-link region of developing cochlear stereocilia and to the periciliary membrane complex of retinal photoreceptors [36,37]. The complex supports hair-bundle maturation in the inner ear and protein trafficking between the inner and outer segments of photoreceptors. *USH2A* is by far the most common cause of USH2, accounting for approximately 70–80% of genetically resolved cases in many populations [38]. Importantly, biallelic *USH2A* variants may cause either syndromic USH2 or nonsyndromic autosomal recessive retinitis pigmentosa. Genotype–phenotype correlations suggest that individuals with two severe or truncating alleles are more likely to develop combined hearing and retinal disease, whereas some hypomorphic alleles may retain sufficient cochlear function and predominantly produce retinal degeneration. Nevertheless, these correlations are not absolute, and marked intrafamilial and interfamilial variability can occur [39].

#### 3.1.3. Usher Syndrome Type 3

Usher syndrome type 3 (USH3) is distinguished by progressive, rather than uniformly congenital, sensorineural hearing loss. Hearing may initially be normal or only mildly impaired but deteriorates over time [40]. Retinal degeneration is also progressive, and vestibular dysfunction is variable. The age at onset and rate of progression differ substantially among patients, making USH3 more difficult to recognize on clinical grounds alone. The only widely accepted canonical USH3 gene is *CLRN1*. *CLRN1* encodes clarin-1, a membrane-associated protein involved in the organization and maintenance of sensory-cell membrane domains, synaptic integrity, and hair-bundle function [41]. Biallelic *CLRN1* variants cause USH3A. The disorder is particularly prevalent in certain founder populations, including individuals of Finnish and Ashkenazi Jewish ancestry, but occurs worldwide. The severity and progression of hearing, retinal, and vestibular manifestations vary according to genotype and genetic background. *HARS1*, encoding cytoplasmic histidyl-tRNA synthetase, was previously proposed as a cause of *USH3B*. However, the evidence supporting *HARS1* as an established Usher syndrome gene remains limited, and it is not generally included among the canonical USH genes in recent stringent classifications.

#### 3.1.4. Atypical Usher Phenotypes

Beyond the classical USH1–USH3 spectrum, combined hearing loss and retinal degeneration may also result from pathogenic variants in genes such as *CEP78*, *CEP250*, *ARSG*, and *ABHD12* [42,43]. These disorders encompass atypical Usher phenotypes, Usher-like disorders, and other syndromic retinal dystrophies accompanied by hearing loss. *CEP78*- and *CEP250*-related disorders are commonly associated with progressive retinal dystrophy and sensorineural hearing loss and have frequently been described as atypical Usher syndromes. Biallelic *ARSG* variants cause a late-onset auditory–retinal disorder that has been proposed as Usher syndrome type 4 (USH4). In contrast, biallelic *ABHD12* variants cause PHARC syndrome, a multisystem neurodegenerative disorder characterized by polyneuropathy, hearing loss, ataxia, retinitis pigmentosa, and cataract. These disorders should therefore be distinguished from the canonical USH1–USH3 syndromes, despite their overlapping auditory and retinal phenotypes. Overall, the considerable genetic and phenotypic heterogeneity of Usher syndrome complicates diagnosis and prognosis [44]. Variants in the same gene may produce classical Usher syndrome, atypical disease, or apparently nonsyndromic hearing or retinal impairment, depending on their molecular consequences and residual protein function.

### 3.2. Heimler Syndrome

Heimler syndrome is a rare autosomal recessive disorder caused predominantly by hypomorphic biallelic variants in the peroxisome-biogenesis genes *PEX1* and *PEX6* [45,46,47,48,49]. Rare patients carrying biallelic *PEX26* variants with a Heimler-like phenotype have also been reported, although the gene–disease evidence is substantially more limited than that for *PEX1* and *PEX6* [47,50,51]. The condition is traditionally classified as Heimler syndrome type 1, caused by *PEX1* variants, and Heimler syndrome type 2, caused by *PEX6* variants. Rather than representing an isolated disease entity, Heimler syndrome is now considered the mildest phenotypic end of the Zellweger spectrum of peroxisome-biogenesis disorders.

PEX1 and PEX6 encode interacting AAA-ATPases that form a heterohexameric complex required for recycling the peroxisomal matrix-protein import receptor PEX5 [50]. Severe loss-of-function variants markedly impair peroxisomal protein import and cause multisystem Zellweger spectrum disorders, whereas Heimler syndrome is generally associated with at least one hypomorphic allele that preserves partial peroxisomal activity. This residual function explains the absence of the profound neurological, hepatic, and craniofacial abnormalities typically observed in severe peroxisomal disease.

The cardinal clinical features include congenital or early-onset bilateral sensorineural hearing loss, amelogenesis imperfecta or enamel hypoplasia of the permanent dentition, and variable nail abnormalities [52]. Retinal involvement is increasingly recognized and may manifest as rod-cone or cone-rod dystrophy, pigmentary retinopathy, macular dystrophy, photoreceptor loss, intraretinal cystoid spaces, and progressive visual-field constriction. However, the ocular phenotype is heterogeneous, and some genetically confirmed patients have no clinically apparent retinal disease at the time of diagnosis [53,54]. Because hearing loss may precede retinal and dental manifestations, affected children can initially be misdiagnosed as having nonsyndromic hereditary deafness or Usher syndrome. Detailed ophthalmologic examination, dental assessment, peroxisomal biochemical testing, and molecular analysis of PEX1, PEX6, and, in selected unresolved cases, PEX26, are therefore important for establishing the diagnosis and guiding longitudinal surveillance.

### 3.3. Norrie Disease

Norrie disease is a rare X-linked recessive disorder caused by hemizygous pathogenic variants in *NDP*, located on chromosome Xp11.4 [55,56,57]. It predominantly affects males, whereas heterozygous female carriers are usually asymptomatic but may occasionally exhibit peripheral retinal vascular abnormalities or, more rarely, clinically significant ocular disease because of skewed X-chromosome inactivation [58,59,60]. *NDP* encodes Norrin, a secreted cystine-knot growth factor that binds to the Frizzled-4 receptor together with the co-receptors LRP5 and TSPAN12, thereby activating canonical β-catenin signaling. This pathway is essential for the development and maintenance of specialized vascular beds and barrier properties in the retina and cochlea [61,62].

The ocular phenotype is typically severe and manifests prenatally or during early infancy. Affected boys commonly present with bilateral retinal dysplasia, incomplete retinal vascularization, retinal folds or detachment, vitreoretinal proliferation, and leukocoria, termed retinal pseudoglioma. Visual function is frequently profoundly impaired at birth or lost during the first months of life. Progressive secondary changes may include vitreous hemorrhage, cataract, anterior synechiae, corneal opacity, glaucoma, and eventual phthisis bulbi [63,64].

In contrast to the congenital ocular phenotype, hearing is frequently normal at birth. Progressive sensorineural hearing loss develops in approximately 80–90% of affected males, although its onset and rate of progression vary considerably. Earlier studies frequently placed the onset in late childhood or adolescence, but longitudinal audiological data have demonstrated detectable abnormalities as early as 3–8 years of age [65]. Hearing loss may initially be mild, fluctuating, asymmetric, or frequency restricted, but it generally progresses toward bilateral, relatively flat, moderate-to-profound sensorineural loss during adolescence or adulthood [66]. Experimental studies indicate that Norrin deficiency primarily disrupts the development and integrity of the stria vascularis and spiral-ligament microvasculature. Subsequent breakdown of the blood–labyrinth barrier, reduction in the endocochlear potential, and deterioration of cochlear homeostasis precede secondary hair-cell loss and auditory dysfunction [67].

Neurological and behavioral manifestations are variable rather than obligatory. In a cohort of 56 patients, cognitive impairment was reported in approximately 28%, autism or autism-like behavior in 27%, seizures in 10%, and peripheral vascular abnormalities in 38% [68]. Earlier estimates that 30–50% of affected individuals have developmental delay or intellectual disability should therefore be interpreted cautiously because of differences in diagnostic definitions, ascertainment methods, and the difficulty of evaluating cognition in individuals with congenital blindness and progressive hearing loss.

### 3.4. Stickler Syndrome

Stickler syndrome (also known as hereditary arthro-ophthalmopathy) is a genetically heterogeneous connective tissue disorder caused by pathogenic variants affecting collagen and extracellular matrix components. Its core clinical features include ocular abnormalities (high myopia, retinal detachment), hearing loss, facial dysmorphism (flat facial profile), and early-onset arthritis [69,70]. The incidence of this condition in newborns is approximately 1 in 7500 [70,71,72], although the true prevalence is likely underestimated because of variable expressivity and incomplete recognition of milder phenotypes.

The molecular basis of Stickler syndrome primarily involves abnormalities of fibrillar collagens and extracellular matrix organization. It is primarily classified into several types based on the pathogenic genes and clinical phenotypes. The three phenotypes caused by autosomal dominant inheritance are respectively due to mutations in *COL2A1*, *COL11A1*, and *COL11A2* [69,73,74]. The majority of cases are caused by heterozygous variants in *COL2A1*, which account for approximately 80% of genetically confirmed cases and are classified as type 1 Stickler syndrome (STL1) [71,75]. *COL2A1* encodes type II collagen, the predominant collagen component of cartilage and the vitreous humor. Consequently, *COL2A1*-associated disease typically demonstrates prominent ocular manifestations, including membranous vitreous degeneration, high myopia, peripheral retinal degeneration, and a markedly increased risk of retinal detachment. Hearing impairment is usually mild to moderate and may result from abnormalities of the middle ear, cochlear structures, or both [69,76].

*COL11A1* variants cause type 2 Stickler syndrome (STL2) and account for a smaller but clinically important proportion of cases [77]. Type XI collagen regulates the assembly and organization of collagen fibrils, and *COL11A1*-associated disease is characterized by a higher frequency and severity of sensorineural hearing loss compared with *COL2A1*-related disease. Ocular manifestations may include congenital cataracts, abnormal vitreous architecture, myopia, and retinal detachment, although the vitreoretinal phenotype is often less severe than that observed in *COL2A1*-related Stickler syndrome [78,79].

*COL11A2* variants cause an autosomal dominant Stickler-like phenotype (referred to as STL3) and are distinct from classic Stickler syndrome because ocular abnormalities are usually absent. Instead, patients primarily present with progressive or congenital hearing loss and skeletal abnormalities, overlapping with otospondylomegaepiphyseal dysplasia (OSMED) [80].

In addition to the dominant forms, rare autosomal recessive Stickler syndromes caused by biallelic variants in *COL9A1*, *COL9A2*, and *COL9A3* have been reported [76,81]. These genes encode collagen IX, a FACIT (fibril-associated collagen with interrupted triple helices) collagen that interacts with collagen II fibrils. *COL9A1*- and *COL9A2*-associated disease commonly presents with high myopia, vitreoretinal abnormalities, progressive sensorineural hearing loss, and epiphyseal abnormalities. *COL9A3* variants have been associated with a milder ocular phenotype, although considerable phenotypic variability exists among affected individuals.

The shared pathogenic mechanism underlying auditory and visual involvement in Stickler syndrome is disruption of collagen-dependent extracellular matrix integrity. In the eye, abnormal collagen composition alters vitreous architecture and retinal adhesion, predisposing patients to retinal degeneration and detachment. In the inner ear, defective collagen organization affects the structural integrity of the tectorial membrane, basilar membrane, and supporting structures of the cochlea, resulting in sensorineural or mixed hearing loss.

## 4. Pathogenic Genes of Comorbid Auditory and Visual Dysfunction

Based on their predominant biological functions, the genes are organized into junctional complexes, Cytoskeletal and Membrane-Associated Mechanisms, mitochondria and metabolism, endoplasmic-reticulum stress and proteostasis, peroxisomal biogenesis, extracellular-matrix abnormalities, signal pathway, development anomaly, and tumor (Table 1). These categories encompass both genes that affect the auditory and visual systems through a shared molecular mechanism and genes whose manifestations arise through organ-specific, allele-dependent, developmental, or secondary structural mechanisms. Although several genes participate in more than one cellular process, the present classification emphasizes the predominant mechanism relevant to combined auditory and visual dysfunction.

### 4.1. Junctional Complexes

#### 4.1.1. USH1C

The *USH1C* gene is located on chromosome 11p15.1 and spans approximately 51 kb, comprising 28 exons, of which eight undergo alternative splicing to generate multiple transcript isoforms [82]. Pathogenic variants in *USH1C* are associated with both Usher syndrome type 1C and autosomal recessive nonsyndromic hearing loss (DFNB18), illustrating allelic heterogeneity. *USH1C* encodes harmonin, a multifunctional scaffold protein containing multiple PDZ domains. Several harmonin isoforms are generated through alternative splicing, allowing interaction with distinct molecular partners in sensory cells [83].

In the retina, harmonin is expressed in photoreceptor cells and participates in the organization of protein complexes involved in photoreceptor maintenance and synaptic function [32]. Through interactions with other Usher proteins, harmonin contributes to the stability of photoreceptor synaptic architecture and the trafficking network required for photoreceptor homeostasis. Disruption of harmonin therefore compromises photoreceptor maintenance and promotes retinal degeneration [32,84].

Thus, *USH1C* contributes to auditory–visual comorbidity through disruption of junction-associated Usher protein complexes, impairing mechanotransduction in cochlear hair cells and photoreceptor homeostasis in the retina.

#### 4.1.2. USH1G

The USH1G gene is one of the smallest genes associated with Usher syndrome, spanning approximately 7 kb and containing three exons, two of which contain protein-coding sequences. Biallelic pathogenic variants in *USH1G* cause Usher syndrome type 1G, whereas rare atypical Usher phenotypes and nonsyndromic hearing loss have also been reported, illustrating allelic and phenotypic heterogeneity [85].

*USH1G* encodes SANS, a 461-amino-acid multifunctional scaffold protein containing three ankyrin repeat domains, a sterile alpha motif (SAM) domain, and a C-terminal PDZ-binding motif. Through these domains, SANS interacts with other Usher proteins, thereby contributing to the assembly and stabilization of junction-associated Usher protein complexes.

In cochlear hair cells, SANS localizes to stereocilia and the tip-link region, where it participates in the organization of the mechanotransduction complex and contributes to actin-cytoskeletal organization required for hair-bundle maturation and maintenance. Loss of SANS disrupts tip-link integrity, stereociliary organization, and mechanotransduction, thereby impairing cochlear hair-cell function [86]. In photoreceptors, SANS contributes to the organization of Usher protein complexes and intracellular trafficking processes required for photoreceptor homeostasis. Its deficiency therefore disrupts photoreceptor homeostasis and may contribute to progressive retinal degeneration [87].

#### 4.1.3. PCDH15

The *PCDH15* gene is one of the largest genes associated with Usher syndrome, spanning approximately 1 Mb and containing multiple alternatively spliced exons that generate several protein isoforms, including long isoforms of nearly 2000 amino acids. Biallelic pathogenic variants in *PCDH15* cause Usher syndrome type 1F (USH1F), whereas hypomorphic variants may result in autosomal recessive nonsyndromic hearing loss (DFNB23), illustrating genotype–phenotype variability [88,89,90].

*PCDH15* encodes protocadherin-15, a member of the cadherin superfamily and a calcium-dependent cell adhesion molecule. In cochlear hair cells, PCDH15 forms the lower component of the CDH23–PCDH15 tip-link complex, which connects adjacent stereocilia and regulates mechanically gated ion-channel activation. Disruption of PCDH15 impairs tip-link formation, stereociliary organization, and mechanotransduction, thereby compromising cochlear hair-cell function [91]. In retinal photoreceptors, PCDH15 contributes to Usher protein-network organization and photoreceptor maintenance, and its deficiency disrupts photoreceptor homeostasis and may contribute to progressive retinal degeneration [89].

#### 4.1.4. CDH23

*CDH23* is one of the most common causes of USH1 after MYO7A and is also responsible for autosomal recessive nonsyndromic hearing loss (DFNB12). Genotype–phenotype correlations indicate that hypomorphic missense variants retaining partial *CDH23* function are frequently associated with isolated hearing loss, whereas severe loss-of-function variants are more commonly associated with *USH1D*, characterized by congenital profound hearing loss, vestibular dysfunction, and progressive retinitis pigmentosa [92,93,94].

*CDH23* spans approximately 300 kb and encodes a large cadherin protein containing multiple extracellular calcium-binding cadherin repeats. In cochlear hair cells, CDH23 forms the upper component of the CDH23–PCDH15 tip-link complex, which connects adjacent stereocilia and is essential for mechanically gated channel activation and normal mechanotransduction [95,96]. Loss of CDH23 disrupts tip-link integrity, leading to abnormal stereociliary organization and impaired cochlear mechanotransduction.

In retinal photoreceptors, *CDH23* is expressed in specialized membrane domains and contributes to photoreceptor structural maintenance and Usher protein network organization. Defective CDH23 function therefore results in progressive photoreceptor degeneration and retinitis pigmentosa [97].

#### 4.1.5. USH2A

Biallelic *USH2A* variants are associated with both Usher syndrome type 2 (USH2) and nonsyndromic autosomal recessive retinitis pigmentosa [98,99]. The *USH2A* gene spans approximately 800 kb and contains 72 exons, generating two major protein isoforms. Isoform a (usherin-short), encoded by the first 21 exons, is a secreted extracellular matrix protein, whereas isoform b (usherin-long), encoded by the full-length transcript, is a large transmembrane protein of 5202 amino acids containing extracellular laminin G and fibronectin type III domains [100].

In cochlear hair cells, usherin forms the USH2 protein complex with ADGRV1 and WHRN at the ankle-link region of developing stereocilia, contributing to stereociliary bundle development and organization [101]. In photoreceptors, long usherin localizes predominantly to the periciliary membrane region, where it participates in the organization of protein-trafficking pathways required for photoreceptor outer-segment homeostasis. Loss of usherin function therefore disrupts the integrity of the USH2 protein network, affecting hair-bundle organization in the cochlea and periciliary homeostasis in retinal photoreceptors [82].

#### 4.1.6. WHRN

The *WHRN* gene encodes whirlin and is associated with both Usher syndrome type 2D (USH2D) and autosomal recessive nonsyndromic hearing loss (DFNB31). Whirlin is a cytoplasmic scaffold protein that interacts with other Usher proteins, including usherin (USH2A) and ADGRV1, through PDZ-mediated interactions [102,103]. In cochlear hair cells, whirlin localizes to stereocilia and regulates hair-bundle elongation, organization, and mechanotransduction complex assembly [104]. In photoreceptors, the long isoform is expressed at the connecting cilium and synaptic regions, where it contributes to photoreceptor structural maintenance and protein trafficking. Loss of whirlin function therefore disrupts Usher protein-complex organization, impairing stereociliary architecture in cochlear hair cells and photoreceptor structural and trafficking homeostasis in the retina [105].

#### 4.1.7. ADGRV1

*ADGRV1*, also known as GPR98 and VLGR1, encodes adhesion G protein-coupled receptor V1, a large transmembrane protein that participates in Usher-associated protein complexes in both cochlear hair cells and retinal photoreceptors. In developing cochlear hair cells, *ADGRV1* localizes predominantly to the ankle-link region at the base of stereocilia, where it interacts with other Usher-associated proteins, including *USH2A*, *WHRN*, and *PDZD7*, and contributes to ankle-link formation and hair-bundle organization. Loss of ADGRV1 disrupts the organization of this protein network, leading to abnormal stereociliary bundle development and impaired hair-cell function.

In retinal photoreceptors, ADGRV1 localizes to the periciliary membrane and connecting-cilium region, where it forms part of the USH2-associated protein network and contributes to the organization of membrane-associated links and protein trafficking between the inner and outer segments. Disruption of ADGRV1 therefore compromises periciliary organization and photoreceptor outer-segment homeostasis. Although ADGRV1 is an adhesion G protein-coupled receptor and additional signaling functions have been proposed, the extent to which canonical GPCR signaling contributes directly to the auditory and retinal phenotypes remains incompletely defined [106,107,108,109,110].

#### 4.1.8. PDZD7

*PDZD7* encodes a PDZ-domain-containing scaffold protein that interacts with other USH2-associated proteins and contributes to the organization and stability of multiprotein complexes at the ankle-link region of developing cochlear stereocilia. Biallelic pathogenic variants in *PDZD7* are associated with autosomal recessive nonsyndromic hearing loss (DFNB57), consistent with an important role of PDZD7 in hair-bundle organization and cochlear mechanosensory function. Unlike the canonical USH2 genes, *PDZD7* has also been reported to act as a genetic modifier or oligogenic contributor in the presence of pathogenic variants in genes such as *USH2A* or *ADGRV1*. In this context, disruption of PDZD7-dependent protein interactions may alter the stability or organization of the USH2 protein network and modify the extent of retinal involvement. Thus, the phenotypic consequences of *PDZD7* variants appear to depend not only on the functional effect of the *PDZD7* alleles themselves but also on the broader genetic background and interactions with other components of the Usher protein network [111,112].

Overall, genes grouped within the junctional-complex category encode adhesion or scaffold proteins that organize interconnected Usher-associated protein networks in the inner ear and retina. In cochlear hair cells, these proteins predominantly contribute to stereociliary organization, tip-link or ankle-link integrity, and mechanotransduction, whereas in retinal photoreceptors they participate in protein-complex organization, trafficking, and cellular homeostasis. Although the precise molecular roles of individual proteins differ between the two sensory organs, disruption of these interconnected complexes provides a common mechanistic basis for combined auditory and visual dysfunction.

### 4.2. Cytoskeletal and Membrane-Associated Mechanisms

#### 4.2.1. MYO7A

Pathogenic *MYO7A* variants can impair auditory and visual function through disruption of actin-dependent molecular-motor and anchoring functions, thereby affecting intracellular trafficking and the organization of specialized sensory-cell structures. *MYO7A* encodes myosin VIIa, an unconventional actin-based molecular motor that transports and anchors specific protein complexes required for the development, maintenance, and function of cochlear hair cells, retinal photoreceptors, and retinal pigment epithelial (RPE) cells [27,113,114]. Because these cell types use MYO7A-dependent processes for distinct cellular functions, *MYO7A* deficiency produces different organ-specific cellular consequences [115].

In cochlear hair cells, mechanosensory stereocilia located at the apical surface convert sound-induced mechanical displacement into electrical signals. The stereociliary bundle requires precise organization of actin filaments and specialized adhesion complexes, including the CDH23-PCDH15 tip-link complex, which directly participates in mechanotransduction channel gating [116,117]. MYO7A is localized at the tips and upper regions of stereocilia, where it interacts with multiple Usher syndrome proteins, including CDH23, PCDH15, harmonin, and SANS [118,119]. MYO7A regulates the trafficking, anchoring, and maturation of these protein complexes, thereby maintaining stereociliary architecture and mechanotransduction [120]. Pathogenic MYO7A variants disrupt these processes, resulting in abnormal stereocilia development, defective tip-link organization, impaired mechanotransduction, thereby compromising cochlear hair-cell function [121].

In the retina, *MYO7A* has important functions in both photoreceptor and RPE cells [121,122]. Photoreceptor outer segments undergo continuous renewal, requiring coordinated intracellular trafficking and efficient processing of shed outer-segment material. MYO7A participates in actin-dependent intracellular trafficking processes associated with photoreceptor function and RPE homeostasis, including melanosome positioning in RPE cells and the intracellular handling of phagocytosed photoreceptor outer-segment material [122]. Loss of MYO7A function therefore disrupts photoreceptor-RPE homeostasis, impairs outer-segment renewal and RPE-associated trafficking processes, and may contribute to progressive photoreceptor degeneration [27,123,124,125].

Therefore, *MYO7A* represents a characteristic example of a shared molecular defect with organ-specific cellular consequences: disruption of an actin-dependent motor and anchoring system primarily affects stereociliary organization and mechanotransduction in the cochlea, while compromising photoreceptor-RPE trafficking and homeostasis in the retina.

#### 4.2.2. CLRN1

*CLRN1* encodes clarin-1, a four-pass transmembrane protein involved in membrane organization, cytoskeletal regulation, and the maintenance of sensory-cell homeostasis [126]. In the inner ear, clarin-1 is expressed in cochlear hair cells and contributes to the structural organization and functional integrity of stereociliary hair bundles. Loss of *CLRN1* disrupts actin-associated hair-bundle organization and impairs mechanoelectrical transduction, thereby compromising cochlear hair-cell function [127]. Certain pathogenic variants may additionally interfere with clarin-1 processing or membrane trafficking, thereby reducing the amount of functional protein reaching the plasma membrane [128]. Clarin-1 has also been implicated in the organization of inner-hair-cell ribbon synapses and auditory afferent signaling [129].

In the retina, the cellular mechanism appears to differ from that in cochlear hair cells. CLRN1 transcripts in adult mouse and human retina are enriched in Müller glial cells rather than photoreceptors [41]. More recent experimental evidence indicates that CLRN1 deficiency disrupts outer-retinal organization and Müller glia–photoreceptor interactions, leading to secondary, non-cell-autonomous photoreceptor dysfunction and degeneration. Re-expression of *clrn1* in Müller glia alleviated photoreceptor degeneration, supporting an important role for Müller glial dysfunction in the retinal phenotype [130]. Thus, *CLRN1*-related auditory and visual dysfunction appears to reflect a shared requirement for clarin-1 in membrane and cytoskeletal organization, with organ-specific consequences involving hair-bundle mechanotransduction and synaptic organization in the cochlea and Müller glia-dependent photoreceptor homeostasis in the retina.

#### 4.2.3. DIAPH1

*DIAPH1* (diaphanous-related formin 1) encodes a member of the formin family of actin-regulatory proteins and functions downstream of Rho-family GTPases to control actin nucleation, elongation, and cytoskeletal remodeling through tightly regulated formin activity. In cochlear hair cells, DIAPH1-dependent actin dynamics contribute to the maintenance of stereociliary architecture and cytoskeletal stability. Heterozygous pathogenic variants that disrupt normal DIAPH1 autoinhibition can alter actin-polymerization activity, thereby impairing stereociliary organization and hair-cell function. In contrast, biallelic loss-of-function variants in *DIAPH1* have been associated with severe neurodevelopmental abnormalities, in which visual impairment may reflect disrupted neuronal development or dysfunction of central visual pathways rather than primary degeneration of retinal photoreceptors [131,132].

#### 4.2.4. ACTG1

ACTG1 encodes cytoplasmic γ-actin, a highly conserved actin isoform that contributes to cytoskeletal organization, cell morphology, and intracellular mechanical stability. Pathogenic variants in ACTG1 cause autosomal dominant nonsyndromic hearing loss (DFNA20/DFNA26) and, in some cases, Baraitser–Winter syndrome type 2 (BRWS2), demonstrating allelic heterogeneity [133]. In cochlear hair cells, γ-actin is a major component of the cortical actin network that maintains stereocilia structure, hair-bundle stability, and mechanotransduction function. Pathogenic missense variants affecting conserved actin domains can alter actin-filament organization and compromise hair-cell integrity, thereby impairing stereociliary stability and cochlear hair-cell function [134]. Distinct pathogenic *ACTG1* variants can also cause BRWS2, in which disruption of actin-cytoskeletal dynamics affects developmental processes in ocular and neural tissues [135].

### 4.3. Mitochondria and Metabolism

#### OPA1

Pathogenic variants in *OPA1* are a major cause of autosomal dominant optic atrophy (ADOA) and can also result in syndromic phenotypes involving the auditory and nervous systems [136,137]. *OPA1* encodes a dynamin-related GTPase localized to the inner mitochondrial membrane that regulates mitochondrial fusion, cristae organization, mtDNA maintenance, and mitochondrial bioenergetic homeostasis [138].

In the retina, OPA1 dysfunction predominantly affects retinal ganglion cells (RGCs), which are particularly vulnerable to impaired mitochondrial dynamics because of their high energetic demands and long axons [139,140]. Disruption of OPA1-mediated mitochondrial fusion and cristae organization compromises mitochondrial respiration, mtDNA stability, and cellular energy homeostasis, thereby increasing the susceptibility of RGCs to degeneration. Many loss-of-function variants act through haploinsufficiency, whereas certain missense variants, particularly those affecting the GTPase domain, may exert dominant-negative effects and are more frequently associated with severe or syndromic phenotypes [141,142,143].

In the auditory system, OPA1 dysfunction can impair mitochondrial homeostasis in metabolically demanding auditory neurons and compromise neural transmission along the auditory pathway. Accordingly, OPA1-associated hearing dysfunction can involve an auditory-neuropathy phenotype rather than primary degeneration of cochlear hair cells [144,145]. Thus, *OPA1*-related auditory and visual dysfunction reflects a shared defect in mitochondrial dynamics and bioenergetic homeostasis, with tissue-specific vulnerability of retinal ganglion cells and auditory neural pathways.

### 4.4. Endoplasmic-Reticulum Stress and Proteostasis

#### 4.4.1. WFS1

Pathogenic WFS1 variants are associated with heterogeneous auditory and visual phenotypes, including progressive sensorineural hearing loss and optic atrophy or other ocular abnormalities [146]. WFS1 encodes wolframin, an integral endoplasmic-reticulum (ER) membrane glycoprotein containing multiple transmembrane domains, with an N-terminal cytoplasmic region and a C-terminal ER-luminal region [147,148,149,150,151]. Biochemical studies demonstrated that wolframin is sensitive to endoglycosidase H and is predominantly localized to the ER [152]. Wolframin contributes to the maintenance of ER homeostasis through regulation of ER stress responses, intracellular Ca^2+^ homeostasis, and protein-processing and quality-control pathways. Dysfunction of wolframin can therefore promote persistent ER stress and disturb proteostatic and calcium homeostasis, increasing the vulnerability of auditory and visual cells to cellular dysfunction and degeneration [152]. These ER-dependent abnormalities provide a mechanistic basis for the combined auditory and visual involvement associated with WFS1 variants.

#### 4.4.2. ATF6

ATF6 (activating transcription factor 6) encodes a type II transmembrane transcription factor that functions as one of the three major sensors of the unfolded protein response (UPR). Under ER stress, ATF6 is transported from the ER to the Golgi apparatus, where it is cleaved to release a cytosolic bZIP transcription factor domain that translocates to the nucleus and induces genes involved in molecular chaperoning, ER-associated degradation, and protein quality control, thereby enhancing ER proteostatic capacity [153].

Biallelic loss-of-function variants in *ATF6* are an established cause of achromatopsia and related cone dysfunction, and recent evidence indicates that progressive sensorineural hearing loss can also occur in some affected individuals [154]. In the retina, impaired ATF6 signaling disrupts cone photoreceptor development and function, highlighting the dependence of cone cells on intact ER proteostasis. In the auditory system, loss of ATF6 activity compromises cochlear protein homeostasis. In *Atf6*-deficient mice, this is accompanied by UPR dysregulation, stereociliary disorganization, and focal outer-hair-cell loss, providing experimental support for the auditory phenotype observed in affected individuals [155]. Together, these findings suggest that defective ATF6-dependent ER stress adaptation and proteostasis represent a shared molecular mechanism underlying the vulnerability of cone photoreceptors and cochlear hair cells.

### 4.5. Peroxisomal Biogenesis

#### 4.5.1. PEX1

*PEX1* is one of the most frequently implicated genes in Zellweger spectrum disorders (ZSD). The central pathogenic mechanism is defective peroxisomal biogenesis and matrix-protein import, which disrupt multiple peroxisomal metabolic pathways, including very-long-chain fatty-acid metabolism and plasmalogen biosynthesis. *PEX1* encodes an AAA-ATPase that forms a heterohexameric complex with PEX6 and provides the ATP-dependent machinery required for recycling the peroxisomal matrix-protein import receptor PEX5. Following cargo delivery, ubiquitinated PEX5 must be extracted from the peroxisomal membrane and returned to the cytosol for subsequent rounds of protein import. Pathogenic variants that impair PEX1 ATPase activity or PEX1–PEX6 complex function disrupt this receptor-recycling process, thereby reducing the import of peroxisomal matrix enzymes and compromising peroxisomal metabolic homeostasis [156,157,158].

Pathogenic PEX1 variants are associated with a broad phenotypic spectrum whose severity is strongly influenced by residual peroxisomal function. Severe loss-of-function alleles generally cause marked impairment of peroxisomal protein import and more severe ZSD, whereas hypomorphic alleles that retain partial PEX1 activity can produce milder phenotypes, including Heimler syndrome type 1 (HMLR1) [159,160,161,162]. In the auditory and visual systems, reduced but persistent peroxisomal dysfunction can disturb lipid metabolism and plasmalogen homeostasis in sensory tissues, providing a molecular basis for the combined cochlear and retinal involvement observed in mild ZSD/Heimler syndrome.

#### 4.5.2. PEX6

*PEX6* encodes an AAA-ATPase that functions together with PEX1 in the peroxisomal receptor-recycling machinery, and pathogenic variants in either gene can cause Zellweger spectrum disorders [163]. PEX6 co-assembles with PEX1 into an ATP-dependent heterohexameric complex that extracts and recycles ubiquitinated PEX5 from the peroxisomal membrane. Disruption of PEX6 function therefore impairs PEX5 recycling and reduces the efficiency of peroxisomal matrix-protein import, leading to secondary disturbances in peroxisomal lipid metabolism and cellular homeostasis [158,163]. Because PEX1 and PEX6 act within the same molecular machinery, their pathogenic mechanisms largely converge on defective receptor recycling and impaired peroxisomal protein import. However, the extent of residual PEX1–PEX6 complex activity may vary according to the underlying variants and contribute to differences in disease severity [164,165,166].

### 4.6. Extracellular-Matrix Abnormalities

#### 4.6.1. COL11A1

Pathogenic *COL11A1* variants are associated with multiple allelic disorders, reflecting substantial genotype–phenotype heterogeneity. *COL11A1* encodes the α1 chain of type XI collagen, a fibrillar collagen that contributes to the regulation of collagen-fibril assembly and extracellular-matrix organization.

In ocular tissues, type XI collagen contributes to the structural organization of the vitreous extracellular matrix. Disruption of COL11A1-dependent collagen organization alters vitreous architecture and extracellular-matrix integrity, thereby compromising the structural environment that supports normal retinal function. In the inner ear, type XI collagen contributes to the organization and mechanical properties of cochlear extracellular-matrix and supporting structures; pathogenic *COL11A1* variants can therefore impair cochlear structural integrity and auditory function [77,167,168,169,170,171,172].

#### 4.6.2. COL2A1

Pathogenic *COL2A1* variants are associated with a broad spectrum of collagen-related disorders, reflecting substantial allelic and phenotypic heterogeneity [173,174,175,176]. *COL2A1* encodes the α1 chain of type II collagen, a major structural component of cartilage and the vitreous extracellular matrix. Disruption of type II collagen structure or abundance impairs collagen-fibril organization and extracellular-matrix integrity.

In the eye, pathogenic *COL2A1* variants alter vitreous architecture, providing the structural basis for the characteristic vitreoretinal abnormalities of *COL2A1*-related Stickler syndrome. In the inner ear, type II collagen is distributed in the spiral ligament, spiral limbus, tectorial membrane, basilar membrane, and other cochlear connective-tissue structures; disruption of this collagen network may therefore compromise cochlear structural and mechanical integrity.

Variant type and location can further influence the molecular consequences and phenotypic severity of COL2A1-related disease. Glycine substitutions within the triple-helical domain frequently exert dominant-negative effects, whereas truncating variants can result in haploinsufficiency and are commonly associated with Stickler syndrome [177,178,179,180,181,182].

### 4.7. Signal Pathway

#### NDP

The *NDP* gene encodes Norrin, a secreted ligand that activates canonical β-catenin signaling through the FZD4-LRP5-TSPAN12 receptor complex. This signaling pathway is essential for retinal angiogenesis, vascular maturation, and maintenance of blood–retinal barrier integrity [61,183]. Pathogenic *NDP* variants can impair Norrin secretion, receptor binding, or downstream β-catenin signaling, thereby disrupting the development and homeostasis of the retinal vasculature [184].

In the auditory system, Norrin signaling also contributes to the development and maintenance of the cochlear lateral-wall vasculature, particularly the stria vascularis and spiral-ligament microvasculature. Loss of Norrin signaling can impair blood–labyrinth barrier integrity, reduce the endocochlear potential, and disturb cochlear ionic and metabolic homeostasis, with secondary effects on sensory-cell function [61,67,185,186]. Thus, *NDP*-related auditory and visual dysfunction reflects disruption of a shared vascular-signaling pathway, with tissue-specific consequences for the retinal and cochlear vascular barriers.

### 4.8. Development Anomaly

#### 4.8.1. GRHL2

GRHL2 (grainyhead-like transcription factor 2) encodes an epithelial transcription factor that regulates epithelial morphogenesis, differentiation, and maintenance of barrier integrity through transcriptional control of genes involved in cell adhesion and tight-junction formation. Pathogenic variants in GRHL2 cause phenotypically diverse disorders, including autosomal dominant nonsyndromic hearing loss (DFNA28), posterior polymorphous corneal dystrophy type 4 (PPCD4), and rare ectodermal dysplasia-related syndromes [187,188,189].

In the inner ear, GRHL2 regulates epithelial gene networks required for cochlear development and maintenance. Heterozygous loss-of-function variants associated with DFNA28 are thought to reduce functional GRHL2 dosage and disrupt cochlear epithelial homeostasis, resulting in progressive postlingual sensorineural hearing loss with variable age at onset and progression [190]. In contrast, PPCD4 is associated with noncoding regulatory variants that increase or ectopically activate GRHL2 expression in corneal endothelial cells. Aberrant GRHL2 expression promotes acquisition of epithelial-like characteristics and disrupts normal corneal endothelial barrier function, thereby contributing to posterior corneal abnormalities and, in more severe cases, visual impairment.

Biallelic loss-of-function variants in GRHL2 can additionally cause ectodermal dysplasia syndromes, further demonstrating the importance of gene dosage and tissue context in determining the phenotypic consequences of GRHL2 dysfunction. Thus, GRHL2-related auditory and ocular abnormalities arise through distinct allele- and tissue-dependent perturbations of epithelial transcriptional programs rather than through a single identical pathogenic mechanism [191].

#### 4.8.2. GDF6

*GDF6* encodes growth differentiation factor 6 (GDF6, also known as BMP13), a secreted member of the bone morphogenetic protein (BMP) family within the TGF-β superfamily. GDF6 signals through BMP receptor complexes, including BMPR1A/ALK3 or BMPR1B/ALK6, and activates the downstream SMAD1/5/8 pathway to regulate developmental gene transcription [192]. During embryogenesis, GDF6 contributes to skeletal and joint patterning, ocular morphogenesis, retinal development, and photoreceptor survival. Pathogenic variants that alter GDF6 expression or signaling activity can therefore produce allele- and tissue-dependent developmental phenotypes involving the skeletal, auditory, and visual systems.

In the auditory system, *GDF6*-related abnormalities are particularly evident in multiple synostoses syndrome type 4, in which altered BMP signaling can promote abnormal ossification and stapes fixation/otosclerosis, thereby producing predominantly structural impairment of sound transmission. Different *GDF6* variants may produce either reduced or increased signaling activity, indicating that both loss of normal ligand function and dysregulated BMP signaling can disturb the development and maintenance of auditory skeletal structures [193,194,195,196].

In the visual system, reduced GDF6 signaling has been associated with a spectrum of ocular developmental abnormalities, including microphthalmia and coloboma, and *GDF6* variants have also been reported in early-onset retinal dystrophy/Leber congenital amaurosis [197,198]. Experimental models show that GDF6 deficiency disrupts ocular patterning and increases retinal cell death, supporting an additional role for GDF6 signaling in photoreceptor survival. Thus, *GDF6*-related auditory and visual abnormalities arise through allele- and tissue-specific developmental mechanisms: auditory involvement may result predominantly from abnormal ossicular or middle-ear structural development, whereas ocular involvement reflects disturbed eye morphogenesis and retinal-cell survival rather than a single shared sensory-cell degenerative pathway [199,200,201].

### 4.9. Tumor

#### NF2

*NF2* represents a mechanistically distinct form of hereditary auditory–visual comorbidity because its sensory manifestations arise predominantly from tumor-mediated structural damage rather than primary degeneration of cochlear hair cells or retinal photoreceptors. Pathogenic variants in *NF2* are the underlying cause of neurofibromatosis type 2. *NF2* encodes Merlin (also known as Schwannomin), an important tumor-suppressor protein. Loss of NF2 function promotes the development of multiple predominantly benign tumors within the nervous system [202]. *NF2* is located on chromosome 22q12.2 and consists of 17 exons. Its pathogenic core mechanism follows the “two-hit” hypothesis. Patients typically inherit one mutated NF2 allele from a parent, and subsequently, the second normal NF2 allele in a particular cell undergoes mutation or deletion due to somatic factors. This results in the complete loss of functional Merlin protein in that cell, leading to abnormal proliferation and tumor formation. A notable feature of *NF2* is “genotype–phenotype correlation”: mutations causing protein truncation (e.g., nonsense and frameshift variants) are generally associated with more severe disease, whereas missense variants, mosaic variants, and some splice-site variants are associated with milder phenotypes [203,204].

Mutations in the *NF2* gene itself do not directly cause congenital deafness or blindness. However, as the disease progresses, it can lead to severe hearing and visual impairments, even complete loss. Its pathogenic mechanism is not through direct destruction of photoreceptor or hair cells, but rather by inducing benign tumors within the cranial cavity and eyes. These tumors, upon growth, compress nerves or critical structures, resulting in functional loss. The deaf-blindness caused by NF2 is not a hereditary sensory and neural degeneration that begins at birth or in early childhood, such as Usher syndrome. Instead, it is a late-onset, progressive disorder. Patients typically have normal or only mild hearing abnormalities in adolescence or early adulthood, with gradual functional loss occurring over time due to tumor growth [204,205].

Bilateral vestibular schwannomas (acoustic neuromas) are the hallmark lesions of NF2 and the primary cause of its impact on hearing. These tumors grow on the eighth cranial nerve, which is responsible for balance and audition. As the tumors enlarge, they continuously compress and damage the auditory nerve, ultimately leading to bilateral, progressive, and asymmetric sensorineural hearing loss. Most patients progress to a stage requiring hearing assistive devices or even complete deafness before middle age. Hearing improvement can often be achieved through surgical intervention in these patients.

The effects of *NF2* on the eyes are more diverse and primarily result in visual impairment through three mechanisms. First, intracranial tumors (e.g., sphenoid wing meningiomas) growing near the optic nerve or optic chiasm can cause decreased vision and visual field defects through compression. Second, posterior subcapsular cataracts are a highly characteristic early ocular manifestation of *NF2* and may present in childhood. Third, small meningiomas or retinal epiretinal membranes within the eye can lead to retinal epiretinal membrane formation and macular involvement, ultimately resulting in macular distortion, metamorphopsia, and decreased vision [203,206].

**Table 1:** Pathogenic genes, mechanisms, and gene therapy status in comorbid auditory and visual dysfunction.

Classification	Pathogenic Mechanism	Genes	Gene Therapy Status
1 Shared pathogenic mechanism causing deafness and blindness	1.1 Junctional complex	*WHRN*	Preclinical research phase (eye) [207]
		*PCDH15*	Preclinical research phase (inner ear and eye) [208,209]
		*USH1C,* *CDH23*	Preclinical research phase (inner ear) [210,211]
		*USH1G,* *ADGRV1, PDZD7*	No gene therapeutic study identified
		*USH2A*	Clinical Research Phase (eye) [212]
	1.2 Cytoskeleton	*MYO7A*	Clinical Research Phase (eye) [213]/Preclinical research phase (inner ear) [214]
		*CLRN1*	Preclinical research phase (inner ear and eye) [129,215,216]
	1.3 Mitochondria and metabolism	*OPA1*	Preclinical research phase (eye) [217]
	1.4 Endoplasmic-reticulum stress and proteostasis	*WFS1*	Preclinical research phase (eye) [149]
		*ATF6*	No gene therapeutic study identified
	1.5 Peroxisome	*PEX1*	Preclinical research phase (eye) [218]
		*PEX6*	No gene therapeutic study identified
	1.6 Extracellular matrix	*COL11A1, COL2A1*	No gene therapeutic study identified
	1.7 Signal pathway	*NDP*	Preclinical research phase (inner ear and eye) [219]
2 Different pathogenic mechanism causing deafness and blindness	Cytoskeletal/Developmental anomaly	*DIAPH1, ACTG1*	No gene therapeutic study identified
3 Developmental abnormalities	Developmental signaling and regulation	*GRHL2, GDF6*	No gene therapeutic study identified
4 Tumor	Tumor	*NF2*	No gene therapeutic study identified

## 5. Gene Therapy for Genetic Deafness–Blindness

Gene therapy for hereditary auditory and visual disorders encompasses several distinct strategies [220,221]. Gene augmentation or replacement aims to deliver a functional copy of a defective gene to the relevant target cells, most commonly using adeno-associated viral vectors. This approach is particularly suitable for recessive loss-of-function disorders in which the affected sensory cells remain structurally preserved. However, the limited packaging capacity of conventional AAV vectors, approximately 4.7 kb, creates a major obstacle for large genes implicated in deaf–blind syndromes, including MYO7A, USH2A, ADGRV1, and CDH23 [222,223,224,225,226,227,228]. To overcome this limitation, dual-AAV systems, truncated functional constructs, lentiviral vectors, and nonviral delivery systems are being explored. However, at present, NDP only has animal models and lacks clinical research [219]. A second major strategy is RNA modulation, particularly antisense oligonucleotide-mediated correction of aberrant pre-mRNA splicing. Unlike gene replacement, this approach does not introduce a complete gene copy or permanently modify genomic DNA. Instead, sequence-specific oligonucleotides bind to pre-mRNA and alter exon recognition, thereby bypassing selected pathogenic variants. This strategy is highly mutation specific, as illustrated by ultevursen, an antisense oligonucleotide designed to induce skipping of exon 13 in USH2A transcripts [229,230]. Additional therapeutic platforms include genome editing, allele-specific gene silencing, RNA interference, nonsense-readthrough approaches, and gene-independent neuroprotective strategies. Although these methods remain largely preclinical for combined auditory and visual disorders, they may ultimately be required for dominant-negative mutations, large genes, or diseases in which simple gene supplementation is insufficient.

A fundamental challenge in treating hereditary deaf-blindness is that the inner ear and retina are anatomically isolated and require different routes of administration. The cochlea is generally accessed through the round-window membrane, cochleostomy, or intracochlear infusion, whereas retinal therapy is commonly delivered through subretinal or intravitreal injection. Consequently, most current programs target either hearing or vision, even when the underlying gene causes disease in both organs. No clinical therapy has yet demonstrated simultaneous restoration of auditory and visual function following a single systemic or local intervention.

### 5.1. Current Status of Gene Therapy in Otolaryngology and Ophthalmology

#### 5.1.1. Gene Therapy for Hereditary Hearing Loss

Clinical translation of inner-ear gene therapy has advanced most rapidly in OTOF-related autosomal recessive deafness 9 (DFNB9). OTOF encodes otoferlin, a protein required for synaptic vesicle exocytosis at the inner-hair-cell ribbon synapse. Patients with biallelic OTOF loss of function variants generally retain structurally intact inner and outer hair cells but cannot efficiently transmit acoustic signals from inner hair cells to auditory nerve fibers [231]. This preserved cellular substrate makes OTOF-related deafness particularly suitable for gene augmentation [232,233,234]. It is worth noting that OTOF-related DFNB9 is a non-syndromic hearing disorder without an established ocular phenotype. It is therefore discussed here as contextual proof of concept for the feasibility and clinical translation of cochlear gene delivery, rather than as direct therapeutic evidence for hereditary auditory–visual comorbidity.

In 2026, Jiang et al. reported a single-arm multicenter trial conducted at eight centers in which 42 participants aged 0.8–32.3 years received AAV1-hOTOF at three vector-dose levels, involving 48 treated ears and follow-up extending to 2.5 years [235]. The investigators classified 38 of 42 participants (90%) and 43 of 48 treated ears (90%) as showing hearing recovery. Among treated ears with hearing recovery, mean auditory brainstem response (ABR) thresholds improved from >97 ± 1 dB nHL at baseline to 54 ± 3, 51 ± 3, 50 ± 3, and 42 ± 5 dB nHL at 1, 1.5, 2, and 2.5 years, respectively, while behavioral audiometry improved from >96 ± 3 dB HL at baseline to 37 ± 5 dB HL at 2.5 years. Speech-perception measures also improved progressively among participants with auditory recovery. Because enrolment was staggered, the number of ears with available long-term ABR data decreased over time, from 27 at 1 year to 17 at 1.5 years, 15 at 2 years, and 7 at 2.5 years. Participants younger than 18 years generally showed greater hearing improvement than adults, and preserved baseline distortion-product otoacoustic emissions and biallelic non-truncating OTOF variants were associated with better auditory outcomes. The primary safety endpoint was dose-limiting toxicity within 6 weeks; no dose-limiting toxicities were observed, although grade 3 adverse events included decreased neutrophil counts.

Separately, the regulatory translation of OTOF gene therapy reached a major milestone on 23 April 2026, when the US Food and Drug Administration granted accelerated approval to Otarmeni (lunsotogene parvec-cwha; formerly DB-OTO) for pediatric and adult patients with severe-to-profound or profound sensorineural hearing loss associated with molecularly confirmed biallelic OTOF variants, preserved outer-hair-cell function, and no previous cochlear implantation in the treated ear [236,237]. Otarmeni is a distinct dual-AAV1 OTOF gene-therapy program from the AAV1-hOTOF study described above. The approval was supported by the CHORD clinical program and represents the first FDA-approved gene therapy for genetic hearing loss. This represents the first approved in vivo gene therapy for a genetic hearing disorder and provides proof of concept that gene augmentation can restore a complex neurosensory function in humans. The success of OTOF demonstrates the feasibility of cochlear gene delivery and long-term monitoring of surgical safety.

#### 5.1.2. Gene Therapy for Inherited Retinal Disease

The eye has historically been a favorable target for gene therapy because of its small volume, relative immune privilege, accessibility for local injection, and availability of high-resolution structural and functional outcome measures. Therapeutic strategies for inherited retinal disease include AAV-mediated gene augmentation, antisense oligonucleotides, genome editing, optogenetics, and cell-based therapy [238,239].

Gene augmentation is currently the most clinically mature strategy and is particularly suitable for recessive loss-of-function disorders in which sufficient target cells remain viable. The landmark example is voretigene neparvovec-rzyl (Luxturna), an AAV2-based therapy approved for patients with confirmed biallelic RPE65 mutation-associated retinal dystrophy and viable retinal tissue [240]. Subretinal delivery of a functional RPE65 copy restores visual-cycle activity and can improve functional vision and retinal light sensitivity, establishing proof of concept for in vivo retinal gene replacement. However, it cannot regenerate photoreceptors that have already been lost, emphasizing the importance of early diagnosis and intervention.

Multiple investigational AAV therapies are being developed for RPGR, GUCY2D, CNGA3, CNGB3, and other IRD genes [241,242,243]. Their efficacy depends on vector design, target-cell transduction, residual retinal structure, treatment age, surgical accuracy, and the sensitivity of clinical endpoints. Large genes, including USH2A and MYO7A, exceed the packaging capacity of conventional AAV vectors and require alternative approaches such as dual-AAV delivery or RNA-based therapy. Antisense oligonucleotides can modify pre-mRNA splicing and are particularly suitable for recurrent splice variants, as illustrated by ultevursen-mediated skipping of USH2A exon 13 [244,245].

Genome editing offers the possibility of permanent correction of pathogenic variants. EDIT-101, an in vivo CRISPR-Cas9 therapy targeting the common intronic CEP290 variant, demonstrated acceptable safety and preliminary improvements in photoreceptor function in a Phase 1–2 study, providing clinical proof of principle for direct retinal genome editing [243]. Nevertheless, off-target effects, irreversible genomic alterations, immune responses, limited editing efficiency, and heterogeneous disease progression remain important concerns [212].

Overall, retinal gene therapy has progressed from experimental proof of concept to approved treatment and multiple clinical programs. However, most therapies remain highly gene- or mutation-specific, require surviving retinal cells, and are limited by vector capacity, delivery-related risks, narrow therapeutic windows, variable outcome measures, and high cost. Comprehensive genetic diagnosis, detailed retinal phenotyping, and long-term safety monitoring are therefore essential for patient selection and clinical implementation.

### 5.2. Gene Therapy of Comorbid Auditory and Visual Dysfunction

#### 5.2.1. Gene Therapy for Usher Syndrome

Usher syndrome is the most common inherited cause of combined sensorineural hearing loss and progressive retinal degeneration. The underlying genes encode proteins involved in stereociliary organization, mechanotransduction, photoreceptor ciliary trafficking, intracellular transport, and synaptic integrity. The therapeutic approach must therefore be tailored not only to the gene but also to the mutation type, residual cell population, disease stage, and affected organ.

At present, clinical development is most advanced for USH2A- and MYO7A-associated retinal disease [82]. However, these programs are directed at preserving or restoring vision and have not been shown to improve hearing.

USH2A is particularly challenging for conventional gene augmentation because its full-length coding sequence is substantially larger than the capacity of a single AAV vector. One strategy is therefore to correct selected mutations at the RNA level. Ultevursen, previously known as QR-421a, is an antisense oligonucleotide developed for patients with retinitis pigmentosa caused by pathogenic variants involving exon 13 of USH2A. It binds to USH2A pre-mRNA and promotes skipping of exon 13 during splicing. Because exon 13 can be removed without disrupting the downstream reading frame, the resulting transcript encodes a shortened usherin protein that may retain partial biological function. The first-in-human Phase 1/2 study evaluated intravitreal ultevursen in patients with USH2A exon 13-associated retinal degeneration. Subsequent Sirius and Celeste studies were initiated to evaluate efficacy in different disease stages; however, the Sirius study was terminated for a business decision rather than a reported safety reason. A new two-year, double-masked, randomized, sham-controlled Phase 2b LUNA study, registered as NCT06627179, was subsequently launched to evaluate repeated intravitreal administration of ultevursen [244,245].

Ultevursen is applicable only to patients carrying eligible pathogenic variants in exon 13. It cannot be extrapolated to all USH2A-associated disease, and it is not expected to restore hearing because intravitreal administration confines treatment to the eye. Its development illustrates both the promise and limitation of precision RNA therapy: a large gene that is difficult to package can be targeted without viral gene replacement, but only a genetically defined subgroup is eligible.

MYO7A causes Usher syndrome type 1B and encodes myosin VIIA, an actin-based molecular motor required for cochlear hair-bundle integrity, photoreceptor-associated transport, and RPE function. The MYO7A coding sequence is too large for a conventional single AAV vector, prompting development of oversized-gene delivery platforms [225,226,246].

An earlier lentiviral product, UshStat/SAR421869, entered a Phase 1/2a subretinal dose-escalation study for USH1B-associated retinitis pigmentosa. The study primarily evaluated safety and potential biological activity, but the lentiviral program did not progress to an established therapy [247,248].

A newer candidate, AAVB-081, uses a dual-AAV strategy in which the MYO7A expression cassette is divided between two vectors [249]. After both vectors transduce the same retinal cell, the two components are designed to reconstitute a functional full-length MYO7A transcript. The Phase 1/2 LUCE-1 trial evaluates a single subretinal injection of AAVB-081 in adults with USH1B-associated retinitis pigmentosa. Enrollment of 15 adult participants was completed in January 2026, with safety, tolerability and preliminary visual efficacy as the principal objectives.

Gene augmentation, editing and RNA-based approaches for USH1C, CDH23, PCDH15, WHRN, and other Usher-associated genes remain predominantly at the preclinical stage [208,210,211].

#### 5.2.2. Gene Therapy for Norrie Disease

Norrie disease is caused by loss-of-function variants in NDP, which encodes the secreted ligand norrin. Norrin activates the FZD4–LRP5–TSPAN12/β-catenin pathway and is essential for retinal vascular development and maintenance of vascular-barrier integrity in both the retina and cochlea. Affected males typically develop severe congenital or very early visual impairment because of abnormal retinal vascularization, whereas progressive sensorineural hearing loss usually appears later in childhood or adolescence.

NDP is particularly attractive as a therapeutic target because norrin is a secreted protein and the delayed onset of hearing loss creates a clinically meaningful intervention window. In a Norrie disease mouse model, systemic AAV9-mediated NDP gene augmentation improved vascular abnormalities in both the retina and cochlea. Neonatal administration rescued both retinal and auditory phenotypes more effectively, whereas treatment at later stages had limited ability to reverse established retinal dysfunction but still reduced or slowed progressive hearing deterioration [219].

These results reveal an important organ-specific therapeutic-window problem. Retinal vascular maldevelopment begins prenatally or very early after birth, meaning that treatment after clinical diagnosis may be too late to reconstruct normal retinal architecture. By contrast, hearing may initially be preserved, allowing postnatal gene therapy to maintain cochlear vascular integrity and prevent secondary hair-cell degeneration.

Despite encouraging preclinical findings, NDP gene therapy has not yet entered a registered human interventional trial. Questions remain regarding vector dose, systemic exposure, immune responses, age at treatment, long-term norrin expression and whether sufficient retinal benefit can be achieved after birth. Accordingly, NDP therapy should be described as a promising preclinical strategy rather than as a treatment already approaching routine clinical use [250,251].

### 5.3. Why Simultaneous Treatment of Hearing and Vision Remains Difficult

The concept of treating a deaf–blind syndrome with a single genetic intervention is biologically attractive but technically challenging. The main barriers include the following.

First, the blood–retinal and blood–labyrinth barriers limit systemic vector access to the relevant target cells. A vector that efficiently reaches photoreceptors may not transduce cochlear hair cells, and vice versa. Second, the two organs have markedly different therapeutic windows. Congenital hearing loss may require treatment during infancy to support auditory-cortex development, whereas retinal degeneration may remain clinically silent for years. Conversely, Norrie disease causes severe prenatal retinal abnormalities but delayed hearing loss. Third, the relevant target cells differ. Usher proteins may need to be restored in cochlear hair cells, retinal photoreceptors and RPE cells, potentially requiring different promoters and vector capsids. Fourth, many deaf–blind genes are exceptionally large. USH2A, ADGRV1, CDH23, PCDH15, and MYO7A cannot be accommodated by a conventional single AAV vector, increasing the complexity of manufacturing and reducing the probability that all vector components enter the same cell. Fifth, established structural degeneration is difficult to reverse. Gene supplementation is most effective when target cells remain alive. Once hair cells, photoreceptors, retinal ganglion cells or auditory neurons are lost, combination approaches involving regeneration, cell replacement or sensory prostheses may be necessary.

Therefore, the near-term clinical model is likely to involve genotype-specific but organ-specific therapy: one product administered to the cochlea and another to the retina, potentially at different ages. A future systemic treatment capable of safely reaching both organs remains an important but unproven goal.

Gene therapy for hereditary auditory–visual comorbidity has entered early clinical translation, but current interventions remain predominantly organ specific. Ultevursen and AAVB-081 target the retinal manifestations of USH2A- and MYO7A-associated Usher syndrome, respectively, whereas NDP gene augmentation has demonstrated dual-organ efficacy only in animal models. Future progress will depend on overcoming large-gene delivery, defining organ-specific therapeutic windows, developing vectors capable of targeting both the retina and inner ear, and integrating genotype-guided treatment with long-term auditory and visual rehabilitation.

## 6. Conclusions

Hereditary auditory–visual comorbidity encompasses disorders arising from shared molecular pathways as well as organ-specific, allele-dependent, developmental, and secondary structural mechanisms. In this review, we summarize 23 genes currently recognized to be associated with combined hearing and visual dysfunction and classify them according to their underlying pathogenic mechanisms, including Usher protein network disruption, cytoskeletal abnormalities, mitochondrial and metabolic dysfunction, endoplasmic reticulum stress, peroxisomal defects, extracellular matrix abnormalities, and developmental signaling disorders. These findings highlight that deafness and blindness are not independent phenotypes but frequently arise from disruption of common molecular processes required for sensory-cell development, maintenance, and homeostasis.

Although advances in gene augmentation, RNA-based therapy, and genome editing have opened new possibilities for treating inherited sensory disorders, current therapeutic approaches remain largely organ-specific and mutation-dependent. Most existing strategies aim to restore either auditory or visual function, while simultaneous recovery of both sensory systems remains an unmet challenge due to differences in anatomical accessibility, delivery routes, disease progression, and therapeutic windows. Future progress will require integrated approaches combining early genetic diagnosis, longitudinal natural-history studies, optimized delivery platforms, and dual-organ precision therapies.

## Data Availability

No new data were created or analyzed in this study. Data sharing is not applicable. All figures in the manuscript were designed by the authors and created using BioRender.com under an appropriate publication license. No previously published third-party figures were reproduced or adapted.
